# Treatment of neuromuscular scoliosis with posterior-only pedicle screw fixation

**DOI:** 10.1186/1749-799X-3-23

**Published:** 2008-06-10

**Authors:** Hitesh N Modi, Seung-Woo Suh, Hae-Ryong Song, Harry M Fernandez, Jae-Hyuk Yang

**Affiliations:** 1 Scoliosis Research Institute, Department of Orthopedics, Korea University Guro Hospital, Seoul, Korea; 2 Rare Disease Institute, Department of Orthopedics, Korea University Guro Hospital, Seoul, Korea

## Abstract

**Background:**

To determine whether posterior-only approach using pedicle screws in neuromuscular scoliosis population adequately addresses the correction of scoliosis and maintains the correction over time.

**Methods:**

Between 2003 and 2006, 26 consecutive patients (7 cerebral palsy, 10 Duchenne muscular dystrophy, 5 spinal muscular atrophy and 4 others) with neuromuscular scoliosis underwent posterior pedicle screw fixation for the deformity. Preoperative, immediate postoperative and final follow-up Cobb's angle and pelvic obliquity were analyzed on radiographs. The average age of the patients was 17.5 years (range, 8–44 years) and the average follow-up was 25 months (18–52 months).

**Results:**

Average Cobb's angle was 78.53° before surgery, 30.70° after surgery (60.9% correction), and 33.06° at final follow-up (57.9% correction) showing significant correction (p < 0.0001). There were 9 patients with curves more than 90° showed an average pre-operative, post operative and final follow up Cobb's angle 105.67°, 52.33° (50.47% correction) and 53.33° (49.53% correction) respectively and 17 patients with curve less than 90° showed average per operative, post operative and final follow up Cobb's angle 64.18, 19.24(70% correction) and 21.41(66.64 correction); which suggests statistically no significant difference in both groups (p = 0.1284). 7 patients underwent Posterior vertebral column resection due to the presence of a rigid curve. The average spinal-pelvic obliquity was 16.27° before surgery, 8.96° after surgery, and 9.27° at final follow-up exhibited significant correction (p < 0.0001). There was 1 poliomyelitis patient who had power grade 3 in lower limbs pre-operatively, developed grade 2 power post-operatively and gradually improved to the pre-operative stage. There was 1 case of deep wound infection and no case of pseud-arthrosis, instrument failures or mortality.

**Conclusion:**

Results indicate that in patients with neuromuscular scoliosis, acceptable amounts of curve correction can be achieved and maintained with posterior-only pedicle screw instrumentation without anterior release procedure.

## Background

The prevalence of severe spinal deformity in patients with neuromuscular disorders is estimated between 50% and 80% [1-3]. The progression of untreated neuromuscular spinal deformities can cause aggravation of pain [4-6], decreased sitting balance [6-10], pressure sores, psychological problems (in patients without mental retardation), compromised pulmonary functions [11,12] and increased mortality [13]. Surgical management has been a reliable option for these patients since introduction of spinal instrumentation by Harrington and subsequent advances by others; most notably by Luque and Cotrel-Dubousset [14,15]. The use of hooks in the thoracic spine has been considered as a gold standard for the treatment of neuromuscular scoliosis. There has been a movement toward the use of thoracic pedicle screws in deformity surgery, based on the reports regarding clinical advantages of pedicle screw fixation in the lumbar spine in terms of enhanced correction and stabilization, when compared with a hook construct [5,16,17]. Uses of Luque rods or unit rod instrumentation have their own disadvantages such as loosening of wires, cutting out of wires, loss of fixation and loss of correction over time and in addition, delayed sitting and ambulation in post-operative phase. Controversy persists, which patient requires anterior release and/or fusion in combination with posterior instrumentation and arthrodesis to improve curve correction.

Treatment of the neuromuscular scoliosis with posterior-only pedicle screw instrumentation is a recent concept, which obviates the need for additional anterior release, and thus risk to life. Main purpose of this study was to evaluate the outcome of single-stage posterior-only pedicle screw instrumentation and fusion for the definitive management of neuromuscular scoliosis. Specific emphasis was kept on correction of scoliosis and maintenance of that correction over time.

## Methods

Between 2003 and 2006, Twenty-six consecutive patients (17 male and 9 female) with progressive neuromuscular scoliosis underwent posterior only pedicle screw fixation and fusion by a single spine surgeon. We did not perform anterior surgery in any of these patients in the study. Seven patients had CP (cerebral palsy), 10 had DMD (Duchenne muscular dystrophy), 5 had SMA (Spinal muscular atrophy) and four (2 post poliomyelitis residual paralysis, 1 each multiple sclerosis and traumatic paraplegia) had other pathologies. Twelve, out of 26 patients, had right side curve and 14 had left side curve. Fifteen, out of the 26 patients, had apex at thoraco-lumbar junction. Fourteen patients had single curves and 12 had double curves. Pre-operatively, 18 patients were wheel chair bound while eight were in ambulatory status (walking independently or with the help of either crutches or walker).

Pre-operative planning of each case was based on a total of eight radiographs, i.e. antero-posterior and lateral radiographs in the sitting and supine positions, lateral bending view and in maximal flexion and extension views. Entire group underwent pulmonary function tests (PFT) preoperatively. We retrospectively reviewed medical records, operative records and sequential radiographs of all patients. Radiographic measurements were recorded in terms of number of curves, amount of preoperative flexibility, preoperative, post-operative and final follow-up Cobb's angle, pelvic obliquity, thoracic kyphosis and lumbar lordosis. Skeletal maturity was assessed by documenting the Risser's sign. Pelvic obliquity was measured as the angle between the line joining two iliac crests and horizontal line. Thoracic kyphosis was measured form upper end-plate of T4 to lower end-plate of T12 and lumbar lordosis was measured form upper end-plate of L1 to lower end-plate of L5. The results of pulmonary function tests, duration of anesthesia, operating time and amount of blood loss were obtained from inpatient charts while postoperative complications and improvement in sitting balance and parents' or care takers' satisfaction were documented from follow up sheets.

### Operative procedure

All patients were operated in prone position with posterior-only approach. Spine was dissected, subperiosteally, up to the tip of the transverse processes at all levels. Pedicle screw inserted bilaterally with free hand technique at all preoperatively decided levels. Bilateral facetectomies was done at all levels,, including the apex, to facilitate the maximum rotational correction. Contouring of the rods were done manually with rod bender and mounted over pedicle screws bilaterally, with concave side being the first followed by the convex. Derotation maneuver was subsequently done, with or without in situ bending of rods, simultaneously on both sides. Rods were fixed by tightening of the caps over screws and posterior fusion achieved, after thorough decortications of posterior laminae, using bone grafts mixed with cancellous allograft. Wound was closed, over two-drainage tube, in layers. The entire patients underwent for radiogram and CT scan postoperatively, which were stored in our computerized PACS system with preoperative data. Posterior vertebral column resection (PVCR) was performed in those patients who had a stiff or rigid spine with a large curve (more than 90°). Pelvic fixation was done using poly-axial ilio-lumbar connectors developed by the authors in patients who had severe pelvic obliquity or contractures in lower extremities.

We have statistically analyzed the results of pre operative and postoperative corrections in Cobb's angle and pelvic obliquity using paired t- test. In addition, we have divided the study group in two categories depending upon the severity of curve: group 1 (curve < 90°) and group 2 (curve > 90°). The correction rate of Cobb's angle and pelvic obliquity between group1 and group 2 were compared using unpaired t-test. P value less than 0.05 was considered the significant for all the tests.

## Results

The average age at the time of operation was 17.5 years (range 8 – 44 years) and the average follow-up was 25 months (range, 18 – 52 months) (table 1). The average percentage of pre operative flexibility was 41% (11%–74%). Average preoperative, postoperative and final follow-up Cobb's angle, pelvic obliquity, thoracic kyphosis and lumbar lordosis are shown in table 2. The average correction rate in Cobb's angle was 60.9% post operatively and 57.9% at final follow up. Similarly, the average correction rate in pelvic obliquity was 44.92% and 43.02% postoperatively and at final follow-up respectively. There has been statistically significant correction achieved, in Cobb's angle (p < 0.0001, paired t-test) and pelvic obliquity (p < 0.0001, paired t-test), post operatively which are maintained at final follow-up. The difference in average preoperative, postoperative and final follow-up thoracic kyphosis did not reveal any significant difference (p = 0.74, ANOVA) while the difference in preoperative, postoperative and final follow-up lumbar lordosis showed significant improvement (p = 0.001, ANOVA).

**Table 1:** Patients' demographics. (Age, diagnosis and level of apex)

**No**	**Age (years)**	**Diagnosis**	**Apex**	**O peration**	**F-U (months)**	**Fixation Level**
1	25	CP	L1	PVCR	25	T5-Pelvis
2	17	CP	L2	C & F	25	T2-Pelvis
3	18	CP	L4	C & F	21	T3-L5
4	16	CP	L1	C & F	22	T4-L4
5	14	CP	T9	C & F	22	T2-L4
6	16	CP	T8	C & F	25	T3-L2
7	20	CP	T10	PVCR	23	T4-L4
8	12	DM D	L2	C & F	52	T3-L5
9	11	DM D	T12	C & F	27	T2-L5
10	8	DM D	L1	C & F	24	T3-L4
11	14	DM D	L2	C & F	24	T4-Pelvis
12	17	DM D	L1	PVCR	26	T3-Pelvis
13	13	DM D	L1	C & F	26	pPelvis
14	15	DM D	L1	C & F	21	T4-L5
15	10	DM D	L1	C & F	21	T2-L5
16	17	DM D	T12	C & F	18	T3-L5
17	14	DM D	T12	C & F	19	T2-Pelvis
18	9	SM A	L1	C & F	33	T3-Pelvis
19	13	SM A	L1	PVCR	31	T3-Pelvis
20	21	SM A	L2	C & F	27	T3-Pelvis
21	10	SM A	L3	C & F	27	T2-Pelvis
22	29	SM A	T12	PVCR	23	T4-L5
23	34	PO LIO	T7	PVCR	22	T3-L4
24	44	PO LIO	L1	PVCR	20	T6-L5
25	13	M S	T8	C & F	25	T3-L5
26	26	PARA	T12	C & F	21	T5-L5

Abbreviations: F-U: follow-up, CP: cerebral palsy, DMD: Duchenne muscular dystrophy, SMA: spinal muscular atrophy, MS: multiple sclerosis, PARA: post traumatic paraplegia, PVCR: posterior vertebral column resection, C & F: correction and fusion.

**Table 2:** Average preoperative, postoperative and final follow-up values for Cobb's angle, pelvic obliquity, thoracic kyphosis and lumbar lordosis.

	**Cobb's angle**			**Pelvic obliquity**			**thoracic kyphosis**			**lumbar lordosis**		
**No.**	**pre-op**	**post-op**	**final**	**pre-op**	**post-op**	**Final**	**pre-op**	**post-op**	**Final**	**pre-op**	**post-op**	**Final**
1	108	76	80	37	23	24	3	16	15	-90	22	20
2	55	19	20	10	3	5	20	16	14	-21	38	44
3	69	10	11	26	6	8	39	27	33	17	29	26
4	52	23	25	7	2	2	33	22	23	40	36	37
5	95	31	30	10	10	8	41	36	33	62	50	38
6	48	14	36	4	1	2	26	10	10	39	32	37
7	94	60	62	6	6	5	30	23	27	34	27	28
8	65	25	24	5	2	2	1	17	20	-4	17	23
9	68	36	35	5	0	3	21	23	22	-38	24	24
10	51	15	16	15	7	9	44	35	33	34	35	36
11	70	21	23	33	12	13	2	18	14	-7	28	24
12	91	45	46	29	12	2	-13	4	3	-79	-15	-15
13	48	2	4	4	1	2	8	25	21	-16	24	23
14	88	32	33	33	20	20	5	3	5	-71	2	5
15	51	7	8	11	4	6	48	38	40	22	38	35
16	109	49	51	32	32	30	8	23	22	3	17	18
17	82	29	28	17	14	15	20	10	12	-40	26	27
18	59	5	6	15	8	10	13	17	16	6	19	20
19	101	26	25	25	14	16	25	24	25	-50	22	23
20	63	33	33	9	3	5	122	65	63	67	45	44
21	84	16	18	7	0	2	7	15	14	-7	28	30
22	123	50	55	40	32	30	31	25	26	38	31	34
23	123	70	68	7	9	7	26	32	33	47	35	39
24	107	64	62	4	2	2	65	29	30	27	37	40
25	55	4	6	6	2	2	34	22	23	6	15	17
26	83	36	38	26	8	11	20	16	15	30	25	26

Note: minus mark in lumbar lordosis indicates lumbar kyphosis and minus mark in thoracic kyphosis indicates thoracic lordosis.

**Figure 1:**
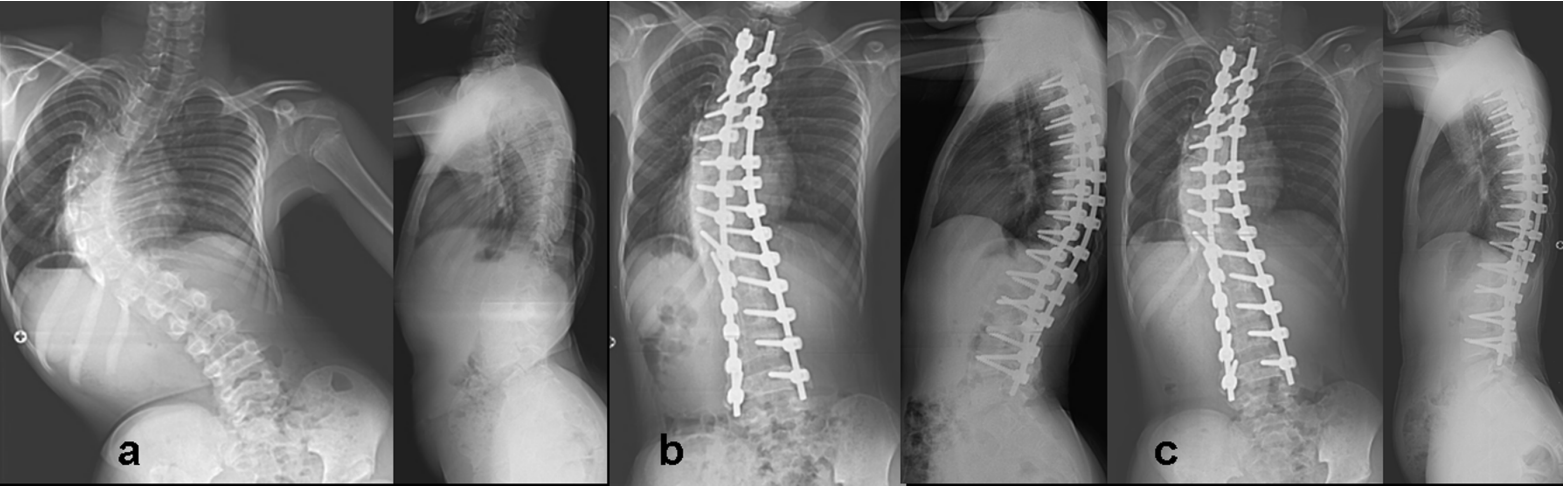
**14 years male with CP**. shows a) preoperative AP and lateral radiogram; b) postoperative AP and lateral radiogram and c) final follow-up AP and lateral radiogram of spine in a fourteen years boy with cerebral palsy. (Patient 5)

**Figure 2:**
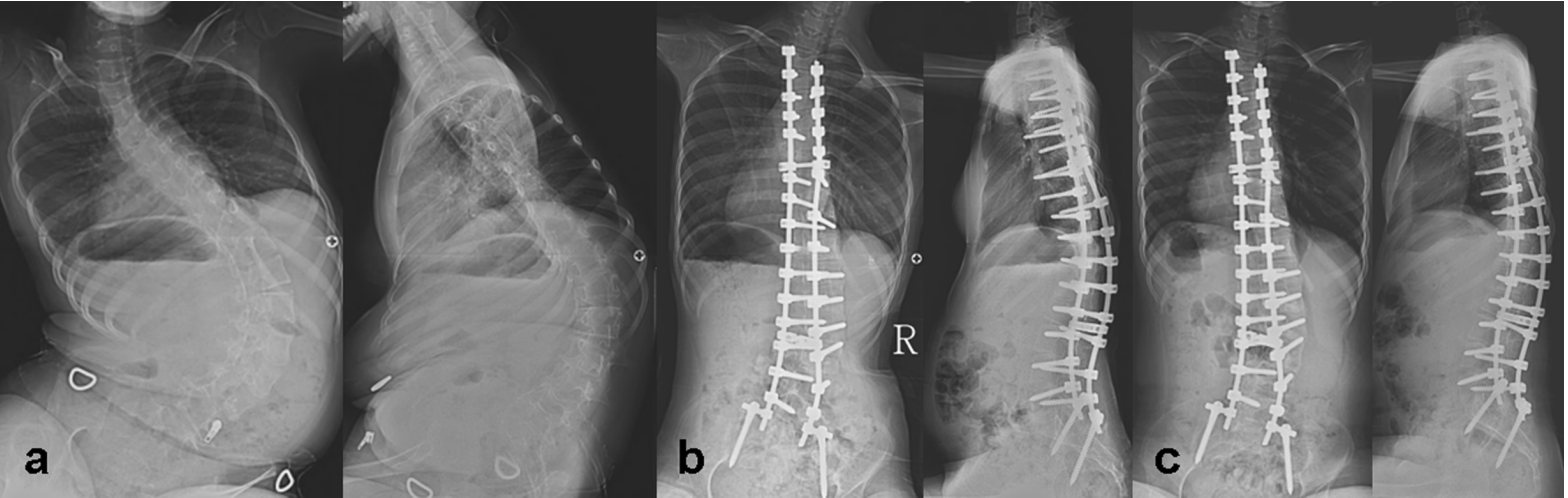
**13 years girl with SMA**. shows a) preoperative AP and lateral radiogram; b) postoperative AP and lateral radiogram and c) final follow-up AP and lateral radiogram of spine in a thirteen years girl with spinal muscular atrophy. (Patient 19)

There were 17 patients with curve less than 90° (group 1) (figure 1) and 9 with curve more than 90° (Group 2) (figure 2) with average pre operative Cobb's angle of 64.18° and 105.67° respectively. Average preoperative pelvic obliquity was 14.94° for group 1 and 18.78° for group 2. The average post operative correction in Cobb's angle was 70.02% and 50.31% for group I and group II respectively; which did not show significant difference (p = 0.1284) in correction between both groups. Similarly, the post operative correction in pelvic obliquity did not reveal any significant difference (p = 0.3239) in correction between both groups. Seven patients underwent PVCR from group 1. Their average pre-operative Cobb angle was 106.71°, with 24% flexibility, had an average post-operative Cobb's angle 55.86° showing 47.65% correction, which is similar to other patients from group 1.

The average number of levels fused was 15. Out of the 26 patients, 23 underwent pulmonary function tests and three patients, all of them being cerebral palsy patients, were non co-operative. Average FVC was 51% (range, 22%–138%), average FEV was 53.5% (range, 15%–147%) and average PEFR was 70.3% (range, 21%–148%). The FVC, FEV1 and PEFR of patients with less than 90° curves were 58%, 63% and 65 % and more than 90° were 38%, 39% and 52% respectively (table 4). Ten patients (4 DMD, 2 CP, 2 SMA, 1 Poliomyelitis, 1 paraplegia) had FVC less than 35% (22%–33%) and six of them needed post-op ventilation. However, we did not perform pulmonary function tests postoperatively.

Abbreviations: WC: wheel-chair bound, WWS: walking without support, Crutch: walking with crutch, walker: walking with walker, PFT: pulmonary function test, PEF: peak expiratory flow, FVC: forced vital capacity, FEV1: forced expiratory volume during first second.

The average operating time was 6 hours 45 minutes (4 hours 30 minutes–10 hours). The average duration of anesthesia was 8 hours 24 min (5 hours 50 minutes–11 hours and 30 minutes). There was not much difference in operating time between the group I (6 hours 7 minutes) and group II (7 hrs 58 min) (table 3). The average intra-operative blood loss was 2773 mililiters (1000–9000 mililiters). The average blood loss for patients who underwent PVCR was 4535 mililiters, whereas it was 2123 mililiters for those who did not undergo PVCR. A total of 13 patients needed postoperative ICU care for an average of 28.3 hours, except one poliomyelitis patient who was in ICU for 2 weeks secondary to DIC and another patient with traumatic paraplegia who had severe bleeding from epidural vessels, was in ICU for 10 days. Six patients required postoperative ventilation (all of them underwent PVCR) for 24 to 36 hours except one poliomyelitis patient who required ventilation for 8 days. The average duration of hospitalization was 24.36 days.

**Table 3:** Values for duration of anesthesia, duration for operation, post operative ICU stay, ventilator support, hospital stay and documentation of infection.

**No**	**Diagnosis**	**Anaes time (Hour:Min)**	**Op time (Hour:Min)**	**EBL (mililiters)**	**ICU (time)**	**Ventilator (time)**	**Infection**	**Hospital Stay (Days)**
1	CP	11:10	10:00	4550	1 Day	NO	NO	32
2	CP	07:30	06:10	1250	NO	NO	NO	24
3	CP	06:50	04:30	1400	NO	NO	NO	30
4	CP	07:40	06:10	2100	NO	NO	NO	14
5	CP	08:00	06:40	1750	NO	NO	NO	26
6	CP	08:00	07:00	1000	NO	NO	NO	10
7	CP	09:00	07:20	5000	3 Days	36 Hours	NO	42
8	DMD	08:10	06:30	2550	NO	NO	NO	24
9	DMD	07:45	05:30	1950	2 Days	NO	NO	15
10	DMD	06:50	04:50	1500	NO	NO	NO	10
11	DMD	07:00	06:00	2100	NO	NO	NO	17
12	DMD	11:35	09:00	4850	3 Days	25 Hours	NO	19
13	DMD	08:40	07:30	2300	5 Hours	NO	NO	22
14	DMD	07:10	05:45	2750	6 Hours	NO	YES	7
15	DMD	06:40	04:50	1750	5.5 Days	NO	NO	18
16	DMD	08:20	06:40	2350	NO	NO	NO	18
17	DMD	08:25	06:10	2300	NO	NO	NO	17
18	SMA	05:50	04:50	1350	3 Hours	NO	NO	31
19	SMA	11:00	08:30	9000	NO	NO	NO	36
20	SMA	10:15	08:15	3750	NO	NO	NO	49
21	SMA	09:40	08:10	2600	NO	NO	NO	19
22	SMA	08:15	06:30	3150	1 Day	26 Hours	NO	23
23	POLIO	10:50	08:50	3100	14 Days	NO	NO	18
24	POLIO	10:10	08:15	2100	1 Day	8 Days	NO	38
25	MS	06:50	05:50	2100	2 Days	NO	NO	15
26	PARA	07:00	06:00	3500	10 Days	24 hours	YES	60

Abbreviations: Anaes time: anaesthesia time, Op time: operation time, EBL: estimated blood loss, ICU: intensive care unit.

Postoperatively all patients exhibited improvement in sitting balance. Two patients who were wheelchair bound preoperatively were able to walk with the help of walker postoperatively and one wheel chair bound patient was able to walk with the help of crutches (table 4). One SMA patient who was able to walk with the help of walker preoperatively was able to walk with the help of crutches postoperatively. None of the patient had deterioration in sitting balance at final follow-up. Parents or care-takers of all patients exhibited better personal and hygienic care postoperatively.

**Table 4:** Pre operative and postoperative ambulatory status with preoperative pulmonary function tests.

		**Walking status**		**pre-operative PFT**		
**No**	**Diagnosis**	**pre-op**	**post-op**	**PEF**	**FVC**	**FEV1**
1	CP	WC	Crutch	FAIL	FAIL	FAIL
2	CP	WWS	WWS	FAIL	FAIL	FAIL
3	CP	WWS	WWS	44	28	28
4	CP	WWS	WWS	121	97	95
5	CP	WWS	WWS	62	51	53
6	CP	WWS	WWS	66	46	Un Co-op
7	CP	WWS	WWS	41	24	25
8	DMD	WC	WC	46	33	29
9	DMD	WC	WC	63	71	74
10	DMD	WC	WC	32	14	15
11	DMD	WC	WC	61	49	53
12	DMD	WC	WC	42	31	32
13	DMD	WC	WC	73	60	61
14	DMD	WC	WC	73	69	72
15	DMD	WC	WC	148	138	147
16	DMD	WC	WC	43	38	41
17	DMD	WC	WC	43	23	21
18	SMA	WWS	WWS	80	91	82
19	SMA	Walker	Crutch	85	64	68
20	SMA	WC	WC	67	31	36
21	SMA	WC	WC	95	89	88
22	SMA	WC	WC	49	22	27
23	POLIO	Crutch	Crutch	51	33	31
24	POLIO	WC	Walker	70	65	63
25	MS	WC	WC	43	37	39
26	PARA	WC	Walker	37	24	28

### Complications

Deep wound infection was seen in one patient with paraplegia who had continuous bleeding from the operated site for which exploration of the wound revealed bleeding from an epidural vessel, which was cauterized. She developed wound dehiscence and deep infection and pus culture grew vancomycin resistant staphylococcus for which she received teicoplanin and regular dressings. One patient with poliomyelitis who had grade 3 power of the lower limbs pre-operatively, developed grade 2 power post-operatively but gradually improved to the pre-operative stage. He also had severe blood loss in the peri-operative period, went into DIC, and was in the ICU for 2 weeks. There was no mortality, pseudarthrosis or implant failurein the study.

## Discussion

Despite the magnitude of this surgery, successful outcome of an operation for spinal deformity, secondary to neuromuscular disease, is considered beneficial by most patients and/or their principal care providers [7,15,18,19]. Aims of the surgery for neuromuscular scoliosis are safe correction of deformity, to stop curve progression, to maintain or recreate sitting balance and to achieve a solid fusion of the balanced spine in the frontal and sagittal planes [20,21]. The average age of the patients in this study was 17.5 years. The increase in age results in increasing stiffness and rigidity of the curve and difficulty to achieve acceptable correction. The mean age in this study was much higher than majority of studies, where patients underwent earlier correction at around 12 years of age or earlier [20,22,23]. However we achieved acceptable correction in both Cobb's angle and pelvic obliquity over al period of 25 months without significant loss of postoperative correction.

We found that there were significant differences in PFT values between curves greater than 90° and less than 90°. Sussman noted an increased risk of postoperative pulmonary problems when the FVC was less than 35% [13,24]. In our study, 10 patients had FVC less than 35% and 6 of them needed postoperative ventilation, supporting Sussman's [25] findings. Postoperative PFT assessment is not being done as a routine basis in our patients. Post-operative PFT values would indicate whether there was any improvement of these parameters following surgery. Therefore, we think, this is the limitation of present study.

Average blood loss for the patients who underwent PVCR was 4535 milliliters, while it was only 2123 milliliters for those who did not undergo PVCR. Other studies [21] with comparable number of fusion levels had average blood loss of 2.4 liters to 2.6 liters in normal fusions and 2.5 liters to 3.4 liters in patients who had associated PVCR. This was the disadvantage of PVCR because it resulted in severe blood loss, and in addition, all of them required postoperative ICU stay and six of the seven patients needed ventilation. Kannan et al [26] comparing blood loss during operation, found that the neuromuscular group had greater blood loss than idiopathic scoliosis.

Average preoperative pelvic obliquity was 16° which decreased to 9° after the surgery, and remained same even at final follow-up. There is a growing controversy regarding the distal extent of fusion in patients with neuromuscular scoliosis (figure 1). There was a general trend to include the pelvis in all cases of neuromuscular scoliosis to correct pelvic obliquity or to prevent its development [20,26-30]. With all the problems described in the literature associated with pelvis fusion [9,31] few patients who had pelvic obliquity greater than 15° and other patients with pelvic obliquity less than 15° had severe lower extremity contractures were chosen for pelvic fusion [32]. Recently, Tsirikos, et al [33,34] challenged the long-term belief that fusion should be avoided in ambulatory patients with CP. In our experience, we have noticed that patients who have gross pelvic obliquity, do not exhibit any problem with sitting balance without much progression over a short term. Therefore presently we do pelvic fixation in patients with pelvic obliquity more than 15° (figure 2) or with severe lower extremity contractures.

Westerlund et al [35] reported 66% correction in Cobb's angle and 75% correction in pelvic obliquity in twenty-six neuromuscular scoliosis with posterior-only unit rod instrumentation. They did not perform any anterior procedure in their series and reported excellent results in immature spine. Boachie-Adjei, et al [4] in their study with 46 patients of neuromuscular scoliosis had an equal number of patients in both the spastic and flaccid group and concluded that corrections for scoliosis and pelvic obliquity were similar in both the groups. In present study, we have also found similar correction in both Cobb's angle and pelvic obliquity without any anterior procedure over a follow-up of 25 months. In addition, none of the patient displayed deterioration in thoracic kyphosis or lumbar lordosis at final follow-up and therefore we did not feel any need for anterior procedure for the correction. Improvements in thoracic kyphosis and lumbar lordosis resulted in to improved sitting balance. Various authors [1,2,36] have used combined anterior and posterior approaches to correct scoliosis, usually in the presence of a very large or stiff curve. Their curves were all less than 90° and they achieved correction rates from 41% to 71%, mostly by using Luque or Unit rod systems. In the group with curve greater than 90° and the group who underwent PVCR, we observed curve correction of 50.31% and 47.65% respectively. To achieve more correction, we prefer PVCR [21] at another extra level. The pedicle screw system has an advantage of being a consolidated fixation including all three columns [5,16,17]. This greatly enhances the ability to simultaneously correct the three dimensional nature of these complex spinal deformities. Using the advantages of both pedicle screw and PVCR, better correction can be expected. Various studies have shown that posterior instrumentation with fusion alone is sufficient to correct and to maintain even larger and stiffer curves in neuromuscular scoliosis and it also prevents crankshaft phenomenon in skeletally immature patients [20,28,37,38]. Reports have also shown that the addition of an anterior procedure, whether staged or same-day, potentially contributes to the risk profile and morbidity in these patients which further supports our criteria of avoiding such measures unless clear benefit can be supported. Our results support that neither anterior release nor anterior arthrodesis is generally indicated to obtain acceptable curve correction in even severe cases.

There are various reports suggesting use of hooks in thoracic level. The main purpose of using hook was to avoid any neurological complications intraoperatively. However other reports [5,16,17] suggested that use of pedicle screw provides stronger purchase and better rotational correction in thoracic spine. Therefore we have used pedicle screws all levels in the subjects and did not notice any major neurological injury postoperatively. Our results are also comparable with the hooks or any other implants.

In present study, all patients underwent for posterior-only pedicle screw and fusion for neuromuscular scoliosis were consecutive and not randomized which is, we think, the limitation of study. If operations had been done in selected patients, results would have been better than this. However, majority of patients achieved acceptable correction in thoracic kyphosis and lumbar lordosis with improvement in sitting balance showed the success of treatment. In addition patients' parents or care takers have also reported better nursing care after operation.

## Conclusion

In conclusion, our series demonstrates the efficacy of posterior-only spinal fusion using the pedicle screw fixation for the definitive management of neuromuscular scoliosis. Curves greater than 90 degrees or rigid curves may require additional PVCR at single level or if necessary, at second level to achieve better outcome. Appropriate care requires the availability of specialized personnel acting as a multidisciplinary team. Our results demonstrate that posterior-only pedicle screw fixation with PVCR if necessary is effective in obtaining and maintaining alignment in the neuromuscular scoliosis population. This technique may avoid those risks incumbent with the addition of an anterior approach.

## Competing interests

The authors declare that they have no competing interests.

## Authors' contributions

HNM has contributed in conception and design and acquisition of data, analysis and interpretation of data, drafting the manuscript and revising it critically, SWS has contributed in conception and design of data, drafting the manuscript and given the final approval of manuscript, HRS has contributed in acquisition of data, revising the manuscript critically and given the final approval, HMF has contributed in drafting the manuscript and designing of data and revising it critically and JHY has contributed in acquisition of data and analysis and interpretation of data.

All authors read and approved the final manuscript.
